# A Generalized ideal observer model for decoding sensory neural responses

**DOI:** 10.3389/fpsyg.2013.00617

**Published:** 2013-09-20

**Authors:** Gopathy Purushothaman, Vivien A. Casagrande

**Affiliations:** ^1^Department of Cell and Developmental Biology, Vanderbilt University Medical SchoolNashville, TN, USA; ^2^Departments of Psychology and Ophthalmalogy and Visual Sciences, Vanderbilt UniversityNashville, TN, USA

**Keywords:** ideal observer model, signal detection theory, neural decoding, receiver operating characteristic, maximum likelihood estimation

## Abstract

We show that many ideal observer models used to decode neural activity can be generalized to a conceptually and analytically simple form. This enables us to study the statistical properties of this class of ideal observer models in a unified manner. We consider in detail the problem of estimating the performance of this class of models. We formulate the problem *de novo* by deriving two equivalent expressions for the performance and introducing the corresponding estimators. We obtain a lower bound on the number of observations (N) required for the estimate of the model performance to lie within a specified confidence interval at a specified confidence level. We show that these estimators are unbiased and consistent, with variance approaching zero at the rate of 1/*N*. We find that the maximum likelihood estimator for the model performance is not guaranteed to be the minimum variance estimator even for some simple parametric forms (e.g., exponential) of the underlying probability distributions. We discuss the application of these results for designing and interpreting neurophysiological experiments that employ specific instances of this ideal observer model.

## Introduction

Ideal observer models are an important tool in the effort to understand the neural bases of perception and behavior (FitzHugh, 1957; Ratliff, 1962; De Valois et al., 1967; Ratliff et al., 1968; Talbot et al., 1968; Barlow and Levick, 1969; Barlow et al., 1971; Mountcastle et al., 1972; Johansson and Vallbo, 1979; Bradley et al., 1987; Newsome et al., 1989; Vogels and Orban, 1990; Geisler, 2001). Ideal observer analysis can be applied to the organism as a whole, as in psychophysical studies, or to a specific stage of information processing within the visual system of the organism, as is often done in neurophysiological studies (sometimes referred to as “sequential ideal observer analysis,” see Geisler, 1989). Here we focus exclusively on ideal observer models that arise in the analysis and interpretation of neurophysiological data. In this context, we define an ideal “observer” model as a set of operations and processes by which the experimenter *optimally* decodes stimuli, perceptual decisions, or behavioral outcomes from sensory neural activity (Green and Swets, 1966; Geisler, 1989, 2001, 2004). In the early stages of a sensory system, such an ideal “observer” model can be used to study the efficiency of a neuron. For example, Barlow et al. (1971) used an ideal detector model to compute detection probability from the number of photons absorbed by photoreceptors and related the results to retinal ganglion cell responses. In this manner, they were able to estimate the average number of impulses emitted by a retinal ganglion cell per quantum of light absorbed by photoreceptors. They concluded ganglion cells are efficient and sensitive. In the intermediate stages of sensorimotor transformation, ideal observer models are often used to optimally decode behavioral choice related information from the responses of a single sensory neuron (Celebrini and Newsome, 1994; Britten et al., 1996). Such analyses associate neural responses with perceptual decisions (rev. Parker and Newsome, 1998). Ideal observer analysis can also be applied to optically imaged cortical signals to assess neural population sensitivity for detection or discrimination (Chen et al., 2006, 2008; Purushothaman et al., 2009; see also rev: Cohen et al., 2011).

The statistical properties of an ideal observer model impact the results. For example, an ideal observer typically yields an unbiased estimate of performance and increasing the number of trials will decrease the variance of this estimate. These assumptions are generally valid when the underlying probability distributions take certain parametric forms but deviations from these assumptions can influence the results. Furthermore, it is not always straightforward to take into account confidence intervals for model performance in interpreting the results. Statistically valid methods of computing confidence intervals are known for some applications (e.g., Agarwal et al., 2005; Sarma et al., 2011) but this is not true in general. Therefore, heuristic or Monte-Carlo simulations are used to compute confidence intervals of ideal observer performance where necessary (e.g., Purushothaman et al., 2009). The main goal of this paper is to investigate the statistical properties and limitations of ideal observer models commonly used in the analyses of neurophysiological data. To achieve this goal, we first generalize four common forms of such ideal observer models.

The first of these was used in studies of the absolute visual detection threshold (Hecht et al., 1942; Hartline et al., 1947; Ratliff, 1962). Hecht et al. (1942) showed that the probability with which human observers detected flashes of light, that presumably delivered a certain average number of quanta of energy (*a*) to the retina, closely followed the probability of drawing a “threshold” number of *n* or more quanta from a Poisson distribution with mean arrival rate *a*. Analysis of the electrophysiological data of Hartline et al. (1947) from the *Limulus* eye showed that the frequency with which a neuron emitted at least a criterion number (*N*_*C*_) of impulses also closely followed the probability of drawing *N*_*C*_ or more impulses from the Poisson distribution with arrival rate equal to *a* (Ratliff, 1962). Implicit in this analysis is the linking hypothesis that the neuron signals to the animal the presence of an external stimulus whenever the number of impulses emitted by the neuron is greater than or equal to *N*_*C*_ (Teller, 1984). Given this hypothesis, the ideal observer model estimates the maximum detection probability for a set of neural responses. It can be said that the criterion *N*_*C*_ is chosen in this model to fit detection probabilities but without regard to the false alarm rate. Since the “maintained” or “background” discharge rate of the neuron also fluctuates (Ratliff et al., 1968; Barlow and Levick, 1969), in some trials, the number of impulses emitted by the neuron will equal or exceed *N*_*C*_ simply due to this random fluctuation and the ideal observer will falsely signal the presence of a stimulus. This false alarm rate is not incorporated into this model.

The second ideal observer model we consider takes the false alarm rate into account [e.g., Barlow and Levick, 1969; rev. Green and Swets, 1966]. Typically, the probability distribution of the number of impulses in the maintained discharge is used to determine *N*_*C*_ so that the probability of false alarm is less than or equal to a predetermined value [e.g., 0.2% in Barlow and Levick (1969)]. The probability distribution for the stimulus-induced response will then determine the detection rate for this criterion. The ideal observer in this analysis performs essentially the same operation as the one above, signaling the presence of a stimulus whenever the number of impulses emitted by the neuron exceeds *N*_*C*_. But this criterion value is chosen based on a constraint on the false alarm rate.

The third model arises in Two-Alternative Forced-Choice (2-AFC) paradigms employed in detection and discrimination studies (Green and Swets, 1966). Typically, a reference and a test stimuli are presented either at two spatial locations (simultaneously) or in two temporal intervals (sequentially). The task of the observer is to indicate the location or the interval in which the test stimulus occurred. Because decisions are based on the comparison of two stimuli or neural responses to two stimuli, there is no need in this case to set a fixed criterion level. For example, the ideal observer can consistently associate the larger response with the test stimulus (e.g., Barlow et al., 1971). Computationally, the experimenter builds two histograms of neural responses, one each for the reference and test stimuli. The correct detection or discrimination probability for the ideal observer in the 2-AFC task is then the average rate at which the observer can correctly identify which sample belongs to which distribution when presented with two random samples, one drawn from the reference distribution and the other from the test distribution (Green and Swets, 1966). This probability can be estimated as the area under the receiver operating characteristic (ROC) curve for the pair of histograms (Green and Swets, 1966).

The fourth model we consider is computationally similar to the third model but has an important conceptual difference in that it is used to predict the choices made by a subject in a 2-AFC task based on the neural responses for near-threshold stimuli (Johansson and Vallbo, 1979; Celebrini and Newsome, 1994; Britten et al., 1996). This analysis can be used to link subjective perceptual decisions to single neuron responses (rev. Parker and Newsome, 1998; Romo, 2001; see also Vallbo and Johansson, 1980). As a consequence, this ideal observer model has found wide application recently (Dodd et al., 2001; Cook and Maunsell, 2002; Romo et al., 2002; Williams et al., 2003; Stoet and Snyder, 2004; Uka and DeAngelis, 2004; Williams et al., 2004; Purushothaman and Bradley, 2005; Pessoa and Padmala, 2005; Gu et al., 2007, 2008; Cohen and Newsome, 2009; Bosking and Maunsell, 2011).

The main difference between the first two ideal observer models and the last two is that the latter models are presented with two observations instead of one, making it possible to render decisions based on a direct comparison of the given observations, independent of a free parameter in the form of a constant criterion number. While this makes the two types of ideal observers different from functional point of view, it is possible to have a single mathematical framework within which the performance of both types of models can be quantitatively described. Consider an ideal observer with two inputs *r*_0_ and *r*_1_ and two outputs *C*_0_ and *C*_1_. Let *P*(*r*_0_) and *P*(*r*_1_) be the probability distributions of the two input variables. In the following, we show that with appropriate choices for *C*_0_, *C*_1_ and *P*(*r*_0_), *P*(*r*_1_), this ideal observer can be used for absolute sensory detection tasks (first two categories described above) as well as for 2-AFC tasks (last two categories). In this framework, the performance (i.e., true positive, false positive, true negative, and false negative rates) of all four types of ideal observers can be described using the same closed-form expression. We then address the following questions: 1) How does the performance of the generalized ideal observer compare to the area under the ROC curve? 2) Is it possible to determine *a priori* the number of input samples required so that the estimated value of the observer's performance will lie within a specified confidence interval at a specified confidence level? 3) Are these estimates unbiased and consistent, i.e., does estimation error decrease with increasing number of observations and at what rate? 4) Do efficient (minimum variance) estimators exist for the performance of these ideal observers? 5) Is the standard method of estimating performance (area under the ROC curve) efficient? Answers to these questions will facilitate a more efficient design of neurophysiological experiments for ideal observer analysis.

## Results

### Generalized ideal observer equations

In the notation introduced above, consider an ideal observer model with inputs *r*_0_ and *r*_1_. Let *S*_0_ and *S*_1_ be the two experimental conditions associated with *r*_0_ and *r*_1_, respectively. The probability distributions *P*_0_(*r*_0_) and *P*_1_(*r*_1_) are given by the conditional distributions *P*_0_(*r*_0_) = *P*_0_(*r*_0_|*S*_0_) and *P*_1_(*r*_1_) = *P*_1_(*r*_1_|*S*_1_). The ideal observer, who has no *a priori* knowledge of which input sample comes from which condition, makes a prediction to that effect using a “decision rule”. If the observer predicts that *r*_0_ comes from the condition *S*_0_ (or, equivalently, from the distribution *P*_0_(*r*_0_|*S*_0_)) and that *r*_1_ comes from *S*_1_ (i.e., from *P*_1_(*r*_1_|*S*_1_)), then the observer will be correct. The opposite association will be incorrect. The variables *r*_0_ and *r*_1_ may represent the frequency of impulses emitted by the neuron. Without loss of generality, assume that the values of *r*_0_ and *r*_1_ lie within the upper right quadrant of the real plane, i.e., the sample space consists of all points $\vec{r}$ = (*r*_0_, *r*_1_) ∈ ℜ^+^ × ℜ^+^. The decision region 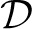 ⊂ ℜ^+^ × ℜ^+^ consists of all values of *r*_0_ and *r*_1_ for which the ideal observer makes a correct prediction. Then the probability of correct prediction for this ideal observer is given by

(1) $$P=\int_{D}p(r_{0},r_{1})d\vec{r},$$

where *p*(*r*_0_, *r*_1_) is the joint probability density function corresponding to the joint probability distribution *P*(*r*_0_, *r*_1_). In many experiments, the responses to the two conditions are independent random variables. Hence *P*(*r*_0_, *r*_1_) = *P*_0_(*r*_0_|*S*_0_)*P*_1_(*r*_1_|*S*_1_). Furthermore, the optimal decision variable (e.g., the likelihood ratio) or its sufficient statistic, involve monotone functions of the two variables *r*_0_ and *r*_1_ thereby resulting in a partition of the sample space ℜ^+^ × ℜ^+^ into a decision region of the form 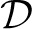 = {(*r*_0_, *r*_1_) ∈ ℜ^+^ × ℜ^+^|*r*_1_ ≥ *r*_0_}. Substituting this integration region into Equation (1) and choosing the summation of the elemental areas along the two possible directions yields two equivalent expressions for the performance of the ideal observer as

(2) $$\begin{array}{l} P\left(p_{0}(r_{0}),p_{1}(r_{1})\right)\overset{\Delta }{=}\int_{0}^{\infty }p_{1}(r_{1})\left\{\int_{0}^{r_{1}}p_{0}(r_{0})dr_{0}\right\}dr_{1}\\ \;=E_{p_{1}}[P_{0}] \end{array}$$

and

(3) $$\begin{array}{l} P\left(p_{0}(r_{0}),p_{1}(r_{1})\right)\overset{\Delta }{=}\int_{0}^{\infty }p_{0}(r_{0})\left\{\int_{r_{0}}^{\infty }p_{1}(r_{1})dr_{1}\right\}dr_{0}\\ \;=1-E_{p_{0}}[P_{1}] \end{array}$$

where *E*_*f*(*x*)_[*G*(*x*)] = ∫*G*(*x*)*f*(*x*)*dx* denotes the expectation of the function *G* with respect to the probability density function *f*, and *p*_*i*_(*r*_*i*_), *i* = 0, 1 are the marginal probability density functions. It is important to note that *P*(*p*_0_(*r*_0_), *p*_1_(*r*_1_)) = 1 − *P*(*p*_1_(*r*_1_), *p*_0_(*r*_0_)) and therefore the order of the two distribution in the argument of *P*(.,.) cannot be exchanged.

This general ideal observer gives rise to the four specific ideal observers described above. In simple detection tasks, the two stimulus conditions are typically *S*_1_ = “Stimulus present” and *S*_0_ = “Stimulus absent.” Choose *p*_0_(*r*_0_) = δ(*r*_0_ − *N*_*C*_) where δ(*x*) is the Dirac delta function such that ∫^∞^_− ∞_δ(*x*) = 1 and δ(*x*) = 0 ∀*x* ≠ 0. Then Equation (2) simplifies to

$$\begin{array}{l} P\left(p_{0}(r_{0}),p_{1}(r_{1})\right)\\ \text{​​​​ }=\int_{0}^{\infty }p_{1}(r_{1})\left\{\int_{0}^{r_{1}}p_{0}(r_{0})dr_{0}\right\}dr_{1}\\ \text{​​​​​ }=\int_{0}^{\infty }p_{1}\text{​}(r_{1})\text{​}\left\{\text{​}\int_{0}^{N_{C}^{-}}\text{​}\delta (r_{0}-N_{C})dr_{0}\text{​}+\text{​}\int_{N_{C}}^{r_{1}}\delta (r_{0}-N_{C})dr_{0}\right\}dr_{1}\text{​​​​}\\ \;=\int_{N_{C}}^{\infty }p_{1}(r_{1})dr_{1} \end{array}$$

which is the probability *P*_1_(*r*_1_ > *N*_*C*_), the hit rate in the detection task. Thus, for the choice of *p*_0_(*r*_0_) = δ(*r*_0_ − *N*_*C*_), the general ideal observer model simplifies to the first category of ideal observers that signal the presence of a stimulus whenever the response of the neuron under consideration equals or exceeds the fixed criterion number *N*_*C*_. The second category of ideal observers used in detection tasks differs from the first only in the choice of the criterion number *N*_*C*_. Therefore these models can be derived using *p*_0_(*r*_0_) = δ(*r*_0_ − *N*_*C*_) where *N*_*C*_ is now determined using the inequality ∫^∞^_*N*_*C*__*p*_0_(*r*_0_)*dr*_0_ ≤ α. It is also clear that the general observer fully describes the third category of ideal observers used to quantify neural detection and discrimination performance in 2-AFC tasks. Finally, for the fourth category of ideal observers, the two “stimulus” conditions need to be replaced with the two “choices” available to the subject. Thus, this general ideal observer provides a complete description of the four types of ideal observers considered above. We should note that this generalization does not imply that all four categories of ideal observers are functionally or physiologically equivalent. This generalization is just mathematical and provides a unified framework for the following analyses.

### Estimators for the performance of the ideal observer

Suppose *R*_1_ = [*r*_11_*r*_12_ … *r*_1*i*_ … *r*_1*N*_] and *R*_0_ = [*r*_01_*r*_02_ … *r*_0*k*_ … *r*_0*N*_] are two sets of *N* samples each obtained in the experiment from the conditions *S*_1_ and *S*_0_, respectively. In the above notation, the elements of *R*_1_ are independent and identically distributed as *P*_1_ and those of *R*_0_ are similarly drawn from *P*_0_. *I*_[0, *x*]_(*y*) is the indicator function of *y* on the closed interval [0, *x*] such that *I*_[0, *x*]_(*y*) = 1 iff *y* ∈ [0, *x*] and 0 otherwise. Then, based on Equation (2), an estimator of the performance of the generalized ideal observer as a function of the samples *R*_0_ for given a value of *r*_1*i*_ can be proposed as

$$\hat{P}(R_{0}|r_{1i})=\frac{1}{N}\sum_{k=1}^{N}I_{\left[0,r_{1i}\right]}(r_{0k}).$$

This provides an estimate for the inner integral in Equation (2), given a value of *r*_1_. Using all 2*N* samples of both *R*_1_ and *R*_0_, P can be estimated as

(4) $$\hat{P}(R_{0},R_{1})=\frac{1}{N^{2}}\sum_{i=1}^{N}\sum_{k=1}^{N}I_{\left[0,r_{1i}\right]}(r_{0k}).$$

The estimator based on Equation (3) can be similarly obtained as

(5) $$\hat{P}(R_{0},R_{1})=\frac{1}{N}\sum_{k=1}^{N}\hat{P}(R_{1}|r_{0k})=\frac{1}{N^{2}}\sum_{k=1}^{N}\sum_{i=1}^{N}I_{\left[r_{0k},\infty \right]}(r_{1i}).$$

Equation (4) provides one simple way to estimate the performance of the generalized ideal observer. We pick one sample from *R*_1_, say *r*_1*i*_, and count the number of samples of *R*_0_ that are less than or equal to *r*_1*i*_. We repeat this for all samples in *R*_1_ and divide the result by *N*^2^. Equation (5) provides a similar method. Computationally, this sequence of operations can be rearranged to resemble the operations involved in computing the area under the ROC curve for the normalized frequency histograms constructed from *R*_0_ and *R*_1_. Thus, there are at least 3 different methods to estimate the performance of this ideal observer. We show below that all three methods compute the area under the ROC curve, empirically constructed from *R*_0_ and *R*_1_.

### Relationship to the area under the ROC curve

For a fixed criterion *T*, the hit rate (β) and false alarm rate (α) are

(6) $$\beta (T)=\int_{T}^{\infty }p_{1}(r_{1})dr_{1}$$

and

(7) $$\alpha (T)=\int_{T}^{\infty }p_{0}(r_{0})dr_{0}.$$

Using Equation (6), we can rewrite the expression for the performance of the ideal observer in Equation (3) as

$$\begin{array}{l} P\left(p_{0}(r_{0}),p_{1}(r_{1})\right)=\int_{0}^{\infty }p_{0}(r_{0})\left\{\int_{r_{0}}^{\infty }p_{1}(r_{1})dr_{1}\right\}dr_{0}\\ \;=\int_{0}^{\infty }p_{0}(r_{0})\beta (r_{0})dr_{0}\\ \;=\int_{0}^{\infty }\beta (r_{0})dP_{0}(r_{0}) \end{array}$$

Using Equation (7) in the above, we have the performance of the ideal observer as

(8) $$P\left(p_{0}(r_{0}),p_{1}(r_{1})\right)=\int_{0}^{\infty }\beta (r_{0})dP_{0}(r_{0})=\int \beta d\alpha .$$

**Figure 1:**
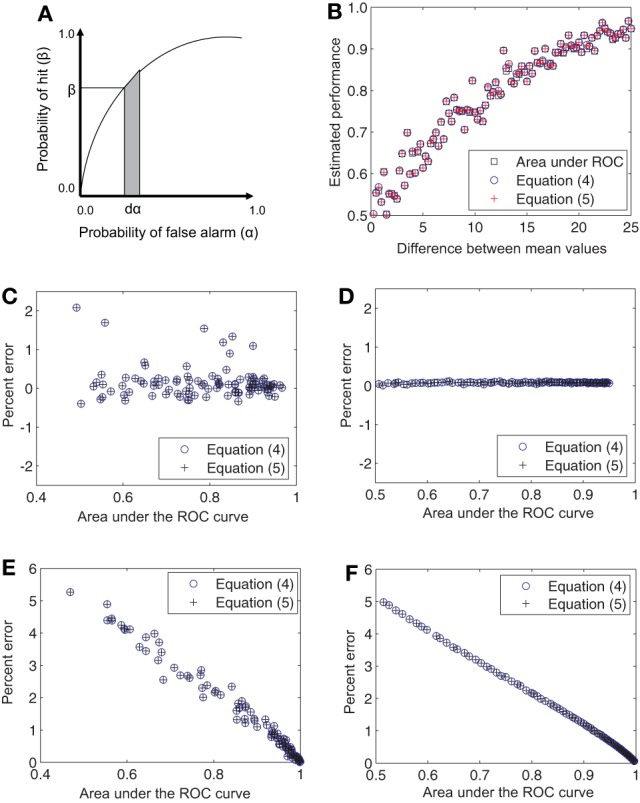
**Relationship of the derived estimators to the area under the ROC curve. (A)** Area under the ROC curve ∫β*d*α has the equivalent definitions given Equations (2) and (3) and admits the estimators given in Equations (4) and (5). **(B)** Single estimates of the area under the ROC curve and Equations (4) and (5) are shown comparatively for a progressively increasing difference in the mean firing rates for Gaussian distributions. The points are predominantly coincident. **(C)** The percent error for single estimates lies within 2%. **(D)** When 100 such trial estimates are averaged together, the percent error falls close to 0%. These differences in the estimates are not systematic and are entirely due to numerical errors. **(E,F)** Same as **(C,D)** but for Poisson distributions. In this case the errors decrease monotonically from about 5 to close to 0%.

Since the ROC curve is the plot of β against α as the criterion varies from 0 to ∞, the quantity ∫ β*d*α is the area under the ROC curve (Figure 1A). Therefore, estimates of the quantities in Equations (2) and (3) are also estimators of the area under the ROC curve.

**Figure 2:**
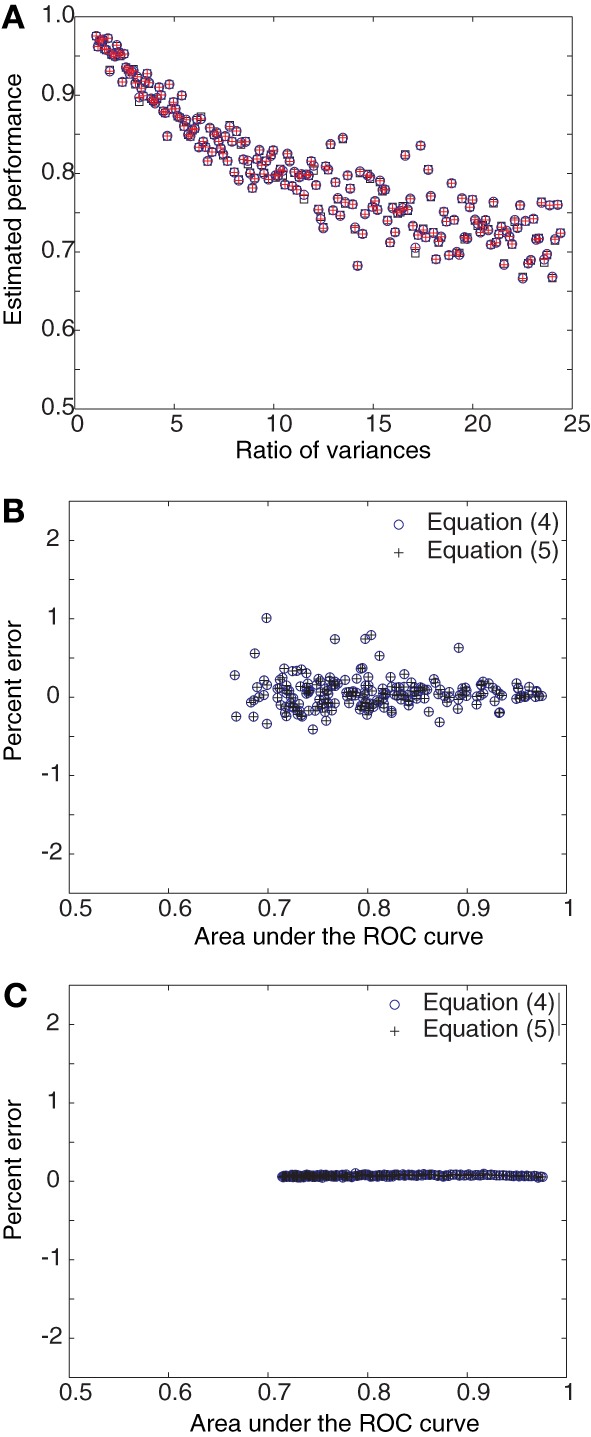
**Derived estimators and area under ROC curve as a function of variances. (A)** Single estimates of the area under the ROC curve and Equations (4) and (5) are compared for progressively increasing ratio of variances for Gaussian distributions. The points are predominantly coincident. **(B)** The percent error for single estimates lies within 2%. **(C)** When 100 such trial estimates are averaged together, the percent error falls close to 0%.

Figures 1, 2 numerically illustrate the fact that estimators (4) and (5) are equivalent to the conventional estimate of performance as the area under the ROC curve. For Figure 1, we assumed Gaussian distributions for *P*_0_ and *P*_1_ with the mean of *P*_1_ greater than that for *P*_0_. A random set of 100 samples were drawn from each distribution and the area under the ROC curve was estimated. Performance was also estimated using Equations (4) and (5). The difference between the mean values of Gaussian distributions was then increased in the range [0.5, 25] in steps of 0.5. The variances were set to 1.28 × mean^1.2^ to mimic the firing rate statistics of MT neurons (Britten et al., 1992; Purushothaman and Bradley, 2005). The estimates were computed for this entire range of mean values (Figure 1B). The deviation of the estimators (4) and (5) from the area under the ROC curve (computed in the traditional manner), was evaluated as Percent error = 100 × (Area under ROC − estimate)/estimate. The three estimates differed by less than 1.5% from each other (Figure 1C). When the estimates were averaged over 100 repetitions, the errors became negligible (Figure 1D). Simulations with Poisson distributions showed errors in the range of 0 − 5% (Figures 1E,F). For Figure 2, we again assumed Gaussian distributions for *P*_0_ and *P*_1_ with the mean of *P*_1_ greater than that for *P*_0_. However, in these simulations, the difference between the mean values were held constant while the ratio of the variance of *P*_1_ to that of *P*_0_ was increased in the range [1, 25]. The percent error was computed as above. These simulations also showed that the estimates averaged over 100 repetitions had negligible error (Figure 2C).

### Unbiased estimation of the ideal observer performance

It is easy to verify that the estimators given in Equations (4) and (5) are unbiased, i.e., their expected values are equal to the true value to be estimated (Van Trees, 1966, pp. 65–73). We note that $\hat{P}$(*R*_0_, *R*_1_) is a joint transformation of the independent random variables *r*_0*k*_ and *r*_1*i*_, *i*, *k* = 1, 2, …, *N* and that *r*_*ik*_, *k* = 1, 2,…, *N* are identically distributed for each *i*. Therefore the expected value of the estimator in Equation (4) can be computed as

(9) $$\begin{array}{c} E[\hat{P}]=\int_{0}^{\infty }\int_{0}^{\infty }\ldots \int_{0}^{\infty }\hat{P}(R_{0},R_{1})\left(\prod_{k=1}^{N}p_{0}(r_{0k})dr_{0k}\right)\left(\prod_{i=1}^{N}p_{1}(r_{1i})dr_{1i}\right). \end{array}$$

Substituting Equation (4) into the above equation, we get

$$\begin{array}{l} E[\hat{P}(R_{0},R_{1})]=\frac{1}{N^{2}}\sum_{i=1}^{N}\sum_{k=1}^{N}\int_{0}^{\infty }p_{1}(r_{1i})\\ \;\left(\int_{0}^{\infty }I_{\left[0,r_{1i}\right]}(r_{0k})p_{0}(r_{0k})dr_{0k}\right)dr_{1i}\\ \;=\frac{1}{N^{2}}\sum_{i=1}^{N}\sum_{k=1}^{N}\int_{0}^{\infty }p_{1}(r_{1i})\left(\int_{0}^{r_{1i}}p_{0}(r_{0k})dr_{0k}\right)dr_{1i}\\ \;=P(P_{0},P_{1}). \end{array}$$

Therefore, $\hat{P}$ in Equation (4) is an unbiased estimator of *P*. Similarly, it can be shown that the estimator of Equation (5) is also unbiased.

### Variance of the estimator

The variances of the estimators in Equations (4) and (5) can be computed by first subtracting *P* from both sides of Equation (4) and squaring them :

(10) $$\begin{array}{l} N^{4}{\left(\hat{P}-P\right)}^{2}\text{​}=\text{​}{\left(\sum_{i=1}^{N}\sum_{k=1}^{N}\left(I_{\left[0,r_{1i}\right]}(r_{0k})-P\right)\right)}^{\text{​}2}\\ \text{ ​}=\text{​}\sum_{i=1}^{N}\sum_{k=1}^{N}{\left(I_{\left[0,r_{1i}\right]}(r_{0k})-P\right)}^{2}+\text{​}\sum_{i=1}^{N}\sum_{k=1}^{N}\sum_{l=1}^{N}\overset{N}{\underset{\begin{array}{c} m=1\\ \left(l,m)\neq (i,\;k\right) \end{array}}{\sum }}\\ \;\left(I_{\left[0,r_{1i}\right]}(r_{0k})-P\right)\left(I_{\left[0,r_{1l}\right]}(r_{0m})-P\right)\\ \text{ ​}=\text{ ​}S_{1}+S_{2} \end{array}$$

Expanding the summand of *S*_1_ as (*I*_[0, *r*_1*i*_]_(*r*_0*k*_) − *P*)^2^ = *I*_[0, *r*_1*i*_]_(*r*_0*k*_) + *P*^2^ − 2 *P I*_[0, *r*_1*i*_]_(*r*_0*k*_) and noting that *E*[*I*_[0, *r*_1*i*_]_(*r*_0*k*_)] = *P*, we obtain for the expectation of the first term, *E*[*S*_1_] = *N*^2^*P*(1 − *P*). Next, we rewrite the second sum as

(11) $$\begin{array}{l} S_{2}=\sum_{i=1}^{N}\sum_{\begin{array}{c} j=1\\ j\neq i \end{array}}^{N}\sum_{k=1}^{N}\left(I_{\left[0,r_{1i}\right]}(r_{0k})-P\right)\left(I_{\left[0,r_{1j}\right]}(r_{0k})-P\right)\\ \;+\sum_{i=1}^{N}\sum_{k=1}^{N}\sum_{\begin{array}{c} m=1\\ m\neq k \end{array}}^{N}\left(I_{\left[0,r_{1i}\right]}(r_{0k})-P\right)\left(I_{\left[0,r_{1i}\right]}(r_{0m})-P\right)\\ \;+\sum_{i=1}^{N}\sum_{k=1}^{N}\sum_{\begin{array}{c} l=1\\ l\neq i \end{array}}^{N}\sum_{\begin{array}{c} m=1\\ m\neq k \end{array}}^{N}\left(I_{\left[0,r_{1i}\right]}(r_{0k})-P\right)\left(I_{\left[0,r_{1l}\right]}(r_{0m})-P\right)\\ \;=S_{21}+S_{22}+S_{23} \end{array}$$

Consider the first sum on the right side of Equation (11) above. We compute the expectation of the product *I*_[0, *r*_1*i*_]_(*r*_0*k*_)*I*_[0, *r*_1*j*_]_(*r*_0*k*_) for *j* ≠ *i* as

(12) $$\begin{array}{c} \begin{array}{l} E\left[I_{\left[0,r_{1i}\right]}(r_{0k})I_{\left[0,r_{1j}\right]}(r_{0k})\right]=\int_{0}^{\infty }\int_{0}^{\infty }p_{1}(r_{1i})p_{1}(r_{1j})\left(\int_{0}^{\infty }I_{\left[0,r_{1i}\right]}(r_{0k})I_{\left[0,r_{1j}\right]}(r_{0k})p_{0}(r_{0k})dr_{0k}\right)dr_{1i}dr_{1j}\\ \;=\int_{0}^{\infty }\int_{0}^{\infty }p_{1}(r_{1i})p_{1}(r_{1j})\left(\int_{0}^{\min(r_{1i},r_{1j})}p_{0}(r_{0k})dr_{0k}\right)dr_{1i}dr_{1j}. \end{array} \end{array}$$

Since min(*r*_1*i*_, *r*_1*j*_) ≤ *r*_1*i*_, we get the following bound:

(13) $$\begin{array}{c} \begin{array}{l} E\left[I_{\left[0,r_{1i}\right]}(r_{0k})I_{\left[0,r_{1j}\right]}(r_{0k})\right]\leq \int_{0}^{\infty }\int_{0}^{\infty }p_{1}(r_{1i})p_{1}(r_{1j})\left(\int_{0}^{r_{1i}}p_{0}(r_{0k})dr_{0k}\right)dr_{1i}dr_{1j}\\ \;=\int_{0}^{\infty }p_{1}(r_{1i})\left(\int_{0}^{r_{1i}}p_{0}(r_{0k})dr_{0k}\right)dr_{1i}=P. \end{array} \end{array}$$

Therefore, we have for the expectation of the first term on the right side of Equation (11) the bound *E*[*S*_21_] ≤ *N*^2^(*N* − 1)[*P*(1 − *P*)]. Now consider the second sum. The expectation of the product *I*_[0, *r*_1*i*_]_(*r*_0*k*_) *I*_[0, *r*_1*i*_]_(*r*_0*m*_) for *m* ≠ *k* is given by

(14) $$\begin{array}{c} \begin{array}{l} E\left[I_{\left[0,r_{1i}\right]}(r_{0k})I_{\left[0,r_{1i}\right]}(r_{0m})\right]=\int_{0}^{\infty }p_{1}(r_{1i})\left(\int_{0}^{\infty }I_{\left[0,r_{1i}\right]}(r_{0k})p_{0}(r_{0k})dr_{0k}\right)\left(\int_{0}^{\infty }I_{\left[0,r_{1i}\right]}(r_{0m})p_{0}(r_{0m})dr_{0m}\right)dr_{1i}\\ \;=\int_{0}^{\infty }p_{1}(r_{1i})\left(\int_{0}^{r_{1i}}\left(r_{0k}\right)p_{0}(r_{0k})dr_{0k}\right)\left(\int_{0}^{r_{1i}}\left(r_{0m}\right)p_{0}(r_{0m})dr_{0m}\right)dr_{1i} \end{array} \end{array}$$

(15) $$\begin{array}{c} \begin{array}{l} \;\leq \int_{0}^{\infty }p_{1}(r_{1i})\;(\int_{0}^{r_{1i}}\left(r_{0k}\right)p_{0}(r_{0k})dr_{0k})dr_{1i}\\ \;=P, \end{array} \end{array}$$

where we used the bound ∫^*r*_1*i*_^_0_(*r*_0*m*_) *p*_0_(*r*_0*m*_)*dr*_0*m*_ ≤ 1 in Equation (14). Therefore, we have for the expectation of *S*_22_ the bound *E*[*S*_22_] ≤ *N*^2^(*N* − 1)[*P*(1 − *P*)]. Finally, we note that in the last term *S*_23_, the summand (*I*_[0, *r*_1*i*_]_(*r*_0*k*_) − *P*)(*I*_[0, *r*_1*l*_]_(*r*_0*m*_) − *P*) is the product of two independent and zero-mean random variables for (*i, k*) ≠ (*l, m*). Hence the variance of the estimator in Equation (4) has the bound

$$\begin{array}{l} E{\left(\hat{P}-P\right)}^{2}\leq P(1-P)\left[\frac{1}{N^{2}}+\frac{2(N-1)}{N^{2}}\right]\\ \;=P(1-P)\left[\frac{2N-1}{N^{2}}\right]. \end{array}$$

Similar calculations yield the same bound for the variance of the estimator in Equation (5).

### Consistency of the estimator

Next, we verify if the estimators are consistent, i.e., if the estimates progressively converge to the true value as the number of observations is increased (Van Trees, 1966, pp. 65–73). To do so, we first apply the Tchebycheff-Bienayme inequality to $\hat{P}$. For any ε > 0, we have

(16) $$\begin{array}{l} \text{Prob}\{|\hat{P}-P|\geq \varepsilon \}\leq \frac{Var(\hat{P})}{\varepsilon^{2}}\\ \;\leq \frac{P(1-P)}{\varepsilon^{2}}\left[\frac{2N-1}{N^{2}}\right]. \end{array}$$

Thus $\hat{P}$ converges to *P* in probability as *N* → ∞ and is a consistent estimator of P.

### Deviation of an estimate from the true value

The above analyses showed that the proposed estimators give an unbiased estimate of the performance of the ideal observer and that as the number of observations increases, the error of estimation (i.e., the variance of the estimator) decreases at the rate of 1/*N*. In addition to establishing these properties, the above analyses also give us tools for designing the ideal observer model. Suppose the experiment has been performed and an estimate of the performance of the ideal observer has been obtained for a neuron. It is desirable to determine the likelihood that the true value of the performance lies within a known range of the estimate obtained, i.e., we would like to state a confidence interval for the estimate at a given significance level. Currently, this confidence interval, when reported, is obtained using bootstrapping or other empirical methods. The above analyses provides a tool for quantifying the deviation of a performance estimate from its true value in a simpler and more rigorous manner. Equation (16) can be used for this purpose. Suppose we require the percent error in the estimate, 100 × |*P* − $\hat{P}$|/$\hat{P}$, to be less than 5%. This gives ε = 0.05 × $\hat{P}$, from which the probability that the true value lies outside this error range can be computed as

(17) $$\begin{array}{l} \text{Prob}\left[100\times \frac{\left|P-\hat{P}\right|}{\hat{P}}\geq 5\%\right]=\text{Prob}\left[|P-\hat{P}|\geq 0.05\times \hat{P}\right]\\ \;\leq \frac{P(1-P)}{{\left(0.05\times \hat{P}\right)}^{2}}\left[\frac{2N-1}{N^{2}}\right]. \end{array}$$

Thus, the quantity $\alpha \overset{\Delta }{=}\frac{P(1-P)}{{\left(0.05\times \hat{P}\right)}^{2}}\left[\frac{2N-1}{N^{2}}\right]$ gives the significance level for the desired confidence interval. We note that since |*P* − $\hat{P}$| ≥ ε, α does not necessarily depend upon the unknown *P*. For large *N*, 2*N* >> 1. Hence α ≈ 2*P*(1 − *P*)/*N*(0.05 × $\hat{P}$)^2^.

**Figure 3:**
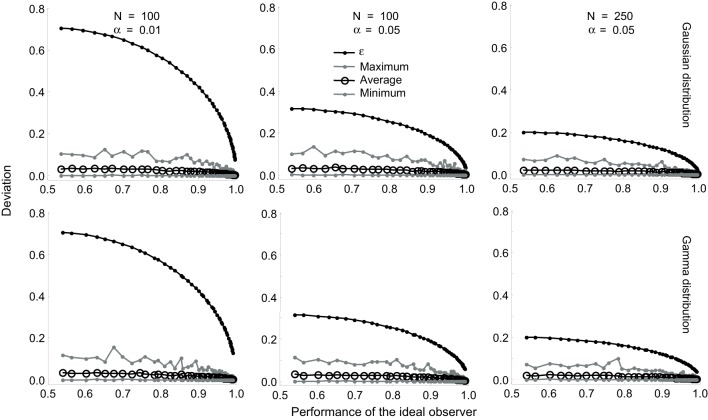
**Tightness of the bound in Equation (17)**. Results are shown for Gaussian (**top row**) and Gamma (**bottom row**) distributions. The difference between the mean values were progressively increased so that the true value of the ideal observer performance varied from 0.5 to 1.0. This performance is plotted on the *X*-axis. The performance was estimated 1000 times and the maximum deviation of the estimate from the true value, the average deviation, and the minimum deviation were computed. The corresponding values of e are also plotted on all the graphs. The effect of varying (α = 0.01 and 0.05) for a fixed (*N* = 100) is shown in the left and middle columns. The effect of varying (*N* = 100 and 250) for a fixed (α = 0.05) is shown in the middle and right columns.

We investigated the tightness of this bound using a series of simulations (Figure 3). We simulated N trials by drawing N samples of *R*_0_ and *R*_1_, each, from Gaussian distributions whose mean values differed by progressively increasing amounts so that the true value of the ideal observer performance varied from 0.5 to 1.0. For each set (*R*_0_, *R*_1_), we obtained one estimate of *P*. We performed this simulation 1000 times and computed the maximum deviation of the estimate from the true value, the average deviation and the minimum deviation for the 1000 estimates. We repeated all of these simulations for Gamma distributions. The results are shown superimposed on the corresponding values of ε for α values of 0.01 and 0.05 (Figure 3). The same pattern of results were obtained for Poisson and scaled Poisson distributions. These simulations show that for small values of *N* (≤ 100) and α(= 0.01), the actual difference between the true and estimated values is much smaller than the theoretical bound ϵ. At α = 0.05 and for higher values of *N*, the theoretical deviation approaches the maximum empirical deviation obtained in the simulations. The implications of the varying tightness of the theoretical bound for experimental design are discussed below.

### Designing experiments for reliable estimation of ideal observer performance

Some previous studies have empirically investigated the number of trials required to obtain a reliable estimate of the ideal observer's performance. For example, Britten et al. (1996) computed “choice probability” separately for odd and even numbered trials. This allowed them to compute a measure of the random dispersion of the probability values. One goal of that investigation was to test whether or not the population average choice probability was significantly different from chance. For the population average choice probability of 0.55, at least 100 trials were required for the odd and even estimates to differ by less than 0.05 (i.e., 0.55–0.5). A different empirical approach was required to estimate the number of trials required to significantly reduce estimation errors in the ROC analysis of optically imaged intrinsic signals (Purushothaman et al., 2009).

From the results obtained in the previous section, we can arrive at a general formula for systematically determining the number trials required for the estimate of the performance of the generalized ideal observer to reach a desired confidence interval. From Equation (16) above, we have, ∀ ε > 0,

(18) $$\text{Prob}\{|\hat{P}-P|\geq \varepsilon \}\leq \frac{P(1-P)}{\varepsilon^{2}}\left[\frac{2N-1}{N^{2}}\right].$$

First, as an example, we consider the Britten et al. (1996) study. Assume that the true value of choice probability in that study was 0.55. Suppose we require that the estimate should lie within ± 0.05 of the true value at an alpha (or significance) level of 0.05, i.e., we require *P*{|$\hat{P}$ − *P*| ≥ 0.05} ≤ 0.05 so that, in concordance with the empirical test performed by Britten et al. (1996), the dispersion in the choice probability estimate reliably excludes the chance value of 0.5. Then the number of trials *N* should be at least $2\sqrt{0.55(1-0.55)/(0.05^{2}\times 0.05)}\approx 89$. The empirical test by Britten et al. (1996) yielded *N* ≈ 100, quite close to this value. However, the above formula also allows us to determine *N* at other significance levels. At a significance level of 0.01, we get *N* ≥ 198.

While many studies that followed Britten et al. (1996) have used this “100 trials” rule to determine *N*, our analysis shows that fewer trials suffice when higher values are expected for the performance of the ideal observer. For example, multistable percepts are linked to fluctuations in neural activity quite strongly (Dodd et al., 2002) and neurons in higher brain areas also show a strong link between their activity and perceptual decisions (Shadlen and Newsome, 2001). Using Table 1 and Equation (18), it is possible to estimate the required value of *N* during experimental design. It is also possible to estimate confidence intervals (i.e., ε) for a given value of *N* during data analysis without resorting to numerical simulations. Table 1 provides a look-up of ε and *N* for various values of *P*. As mentioned above, our simulations showed that at a given value of *N* and α, the actual deviation between the true and estimated values was much smaller than the theoretical bound set at ϵ (Figure 1). Therefore, the values of *N* shown in Table 1 are likely to be overestimates, i.e., fewer trials might suffice to reach the desired confidence interval in some cases.

**Table 1:** **The confidence interval (ε) and the number of trials (*N*) are shown for various true values of *P***.

***P***	**ε**	***N***	
		**α = 0.01**	**α = 0.05**
0.525	10% of *P*	96	43
0.550	10% of *P*	91	41
0.575	10% of *P*	86	38
0.6	10% of *P*	82	37
0.525	5% of *P*	191	85
0.550	5% of *P*	181	81
0.575	5% of *P*	172	77
0.6	5% of *P*	164	73
0.625	10% of *P*	78	35
0.650	10% of *P*	73	33
0.675	10% of *P*	70	31
0.7	10% of I	66	29
0.625	5% of *P*	155	70
0.650	5% of *P*	146	65
0.675	5% of *P*	139	63
0.7	5% of *P*	131	59

The expression for obtaining the number of trials required to reach a given confidence interval ε at a significance level α is $N\approx \sqrt{\frac{P(1-P)}{\varepsilon^{2}\times \alpha }}$. Alternatively, for given values *N* and α, the confidence interval can be computed as $\varepsilon \approx \sqrt{\frac{P(1-P)}{N^{2}\times \alpha }}$.

### Efficient estimators of ideal observer performance may not exist

Since the performance of the ideal observer can be estimated in more than one way, it is natural to ask if some of these methods are “better” than others. In addition to requiring that estimators be unbiased and consistent, it is also required that estimators should be “efficient” when possible (Van Trees, 1966, pp. 66–73). An efficient estimator has the minimum possible variance among all unbiased estimators for a quantity and therefore will yield the lowest possible error for a given number of observations, on average. Under some conditions, maximum likelihood (ML) estimators are minimum variance estimators. Therefore, it is natural to seek for ML estimators for the performance of the ideal observer model. In this section, we first show that $\hat{P}$(*R*_0_, *R*_1_) is “efficient” in a limited sense. We then present a counter-example to show that the maximum-likelihood (ML) estimator for the performance of an ideal observer is not guaranteed to be minimum variance.

We will first describe a limited sense in which $\hat{P}$ is efficient. Let *M*(*R*_0_, *R*_1_) = ∑^*N*^_*i* = 1_ ∑^*N*^_*k* = 1_ *I*_[0, *r*_1*i*_]_(*r*_0*k*_) so that $\hat{P}$(*R*_0_, *R*_1_) = *M*(*R*_0_, *R*_1_)/*N*^2^. Then, for a given value of *P*, the probability distribution function for *M* is simply the binomial distribution

(19) $$P_{M}(M(R_{0},R_{1})=m|P)=\left(\begin{array}{c} N^{2}\\ m \end{array}\right){\left(P\right)}^{m}{\left(1-P\right)}^{N^{2}-m}.$$

Therefore it can be verified that the calculation

$$\frac{\partial \;\log P_{M}(m|P)}{\partial \;P}{\overset{|}{|}}_{P={\hat{P}}_{ml}(m)}=0$$

gives the ML estimator as

(20) $${\hat{P}}_{ml}(m)=\frac{m}{N^{2}}.$$

We note also that

$$\begin{array}{l} \frac{\partial \log P_{M}(m|P)}{\partial P}=\left(\frac{N^{2}}{P(1-P)}\right)\left(\frac{m}{N^{2}}-P\right)\\ \;=\left(\frac{N^{2}}{P(1-P)}\right)\left({\hat{P}}_{ml}(m)-P\right), \end{array}$$

i.e., $\hat{P}$_*ml*_(*m*) satisfies the sufficient condition to be an efficient estimator (Van Trees, 1966, pp. 66–73). In addition, *E*_*M*_($\hat{P}$_*ml*_(*m*)) = *P*. Therefore, $\hat{P}$_*ml*_(*m*) = *m*/*N*^2^ is an unbiased and efficient estimator of *P*. However, it is important to note that $\hat{P}$_*ml*_(*m*) is an estimator of *P* as a function of the transformed random variable *M*(*R*_0_, *R*_1_) and not as a function of *R*_0_ and *R*_1_. The following counter-example shows that it is not possible to guarantee that ML estimators of *P* are minimum variance.

Let the two conditional distributions be exponential, with *p*_0_(*r*_0_) = α_0_ exp(−α_0_ *r*_0_) and *p*_1_(*r*_1_) = α_1_ exp(−α_1_ *r*_1_). We can calculate *P* for this case using Equation (2) as *P*(*P*_0_, *P*_1_) = α_0_/(α_1_ + α_0_). Let us note that

1. if α_1_ = α_0_, then *P* = 0.5,
2. *P* → 1 as α_0_ → ∞ for a given α_1_ < ∞ (i.e., as the mass in the tail of the distribution *P*_0_ accumulates while that of *P*_1_ remains constant), and
3. *P* → 0 as α_0_ → 0 for a given α_1_ < ∞.

Thus the conditional density of the observed variables for a given value of *P* can be written as

$$p(r_{0},r_{1}|P)=\alpha_{0}\alpha_{1}\exp\left[-\left(\alpha_{1}\frac{P}{1-P}r_{0}+\alpha_{0}\frac{1-P}{P}r_{1}\right)\right],$$

which gives

(21) $$\frac{\partial \log p(r_{0},r_{1}|P)}{\partial P}=\frac{\alpha_{0}r_{1}}{P^{2}}-\frac{\alpha_{1}r_{0}}{{\left(1-P\right)}^{2}}.$$

Equating the right hand side to 0, we obtain the ML estimator for *P* in this case as

$${\hat{P}}_{ml}(r_{0},r_{1})=\frac{1}{1+\sqrt{\left(\alpha_{1}r_{0}/\alpha_{0}r_{1}\right)}}.$$

We now note that equation (21) cannot be put in the form

$$\frac{\partial \log p(r_{0},r_{1}|P)}{\partial P}=T(P)[{\hat{P}}_{ml}(r_{0},r_{1})-P],$$

where *T*(*P*) is a function of *P* alone. Therefore, the sufficient condition for $\hat{P}$_*ml*_(*r*_0_, *r*_1_) to be efficient is not satisfied (e.g., Van Trees, 1966, pp. 66–73). Further, it is also clear that $\hat{P}$(*r*_0_, *r*_1_) is a biased estimator. Hence the ML estimator of *P* for this case cannot be guaranteed to be minimum variance.

## Discussion

We proposed a general form of an ideal observer for decoding stimulus information and perceptual decisions from neural responses. We showed that several ideal observer models used in previous studies are special cases of this general form. We investigated the statistical properties of this general ideal observer model. These analyses provide various tools for designing experiments with the goal of using an ideal observer analysis on neural data. We have provided a lower bound on the number of observations required for the estimate to lie within a pre-specified range of its true value (“confidence interval”), within a specified confidence level.

We also showed that there is not a uniformly “best” (i.e., minimum variance) estimator for the performance of the ideal observer since the existence of such an estimator depends on the parametric forms of the underlying probability distributions. It is sometimes argued that computing the area under the ROC curve offers a non-parametric way of estimating ideal observer performance. While it is true that this estimation *procedure* does not depend on the parametric forms of the underlying probability distributions, it is important to note that the resulting *estimate* will be invariably influenced by the underlying parametric forms. Therefore, for some parametric forms and under some conditions, neither the estimators provided in Equations (4) and (5) nor the area under the ROC curve will be efficient. However, regardless of which estimator is chosen, the relationship between the number of trials, the confidence interval and the confidence level derived in this paper can be used to design the experiment and validate the results.

It is worth noting that the number of trials required for the estimate to lie within a confidence interval at a given confidence level is not the *optimum* number of trials required for reaching the decision. Therefore in certain applications other methods, such as sequential probablity ratio tests, may be more appropriate (Wald, 1945).

### Conflict of interest statement

The authors declare that the research was conducted in the absence of any commercial or financial relationships that could be construed as a potential conflict of interest.
